# Soybean α-Amylase Gene Family: Structure and Expression in Response to Abiotic Stresses

**DOI:** 10.3390/ijms27177915

**Published:** 2026-09-04

**Authors:** Mikhail A. Filyushin, Anna V. Shchennikova, Ksenia O. Shilova, Elena Z. Kochieva, Mikhail G. Divashuk

**Affiliations:** 1 Institute of Bioengineering, Research Center of Biotechnology, Russian Academy of Sciences, Leninsky Ave. 33, Bld. 2, 119071 Moscow, Russia; shchennikova@yandex.ru (A.V.S.); ekochieva@yandex.ru (E.Z.K.); 2 All-Russia Research Institute of Agricultural Biotechnology, Timiryazevskaya Str. 42, 127550 Moscow, Russia; ksushilova@gmail.com (K.O.S.); divashuk@gmail.com (M.G.D.)

**Keywords:** soybean, *Glycine max* L., α-amylase genes, gene structure, gene expression, abiotic stress response

## Abstract

α-Amylases are involved in starch breakdown, thereby influencing plant development. Information on the α-amylase genes in soybean is limited. Here, we identified five soybean α-amylase genes from subfamilies AtAMY1 (*GmaAMY5*), AtAMY2 (*GmaAMY4*), AtAMY3 (*GmaAMY1*, *GmaAMY3*), and AMY6 (*GmaAMY2*). In silico analysis indicated that all five genes were actively expressed in leaves, flowers, and pods but weakly in roots. *GmaAMY1–GmaAMY5* mRNAs were predicted to be targets of miRNAs associated with stress response, organ development, and nitrogen fixation. Putative GmaAMY1–GmaAMY5 proteins contained α-amylase-specific catalytic domain, signatures, and active sites. Short-term abiotic stresses (100 mM NaCl, 2.5–20% PEG, and 4 °C cold) applied to the cv. Doka affected both *GmaAMY1–GmaAMY5* expression and the content of starch and soluble sugars in leaves. *GmaAMY1* gene expression increased in response to NaCl and PEG, *GmaAMY2* in response to PEG, and *GmaAMY5* in response to NaCl. Salt stress suppressed the expression of the *GmaAMY2–GmaAMY4* genes. The mRNA levels of all five genes increased after 2 h of cold exposure. Under salinity stress, there was inverse correlation of starch content with *GmaAMY4* expression (r = −0.5135, *p* = 0.0293) and overall *GmaAMY1–GmaAMY5* expression (r = −0.6318, *p* = 0.0049), suggesting a possible role of *GmaAMY* genes in protecting soybean from salinity by maintaining the starch/soluble sugars balance. Our results may aid in the breeding of stress-tolerant soybean varieties.

## 1. Introduction

The main reserve carbohydrate in plants is starch, a polysaccharide that provides energy for plant growth and development, including seed germination [1]. Starch is stored in granules composed of glucose polymers, primarily branched amylopectin organized into semicrystalline structures. The process of amylopectin degradation involves synergistic activity of many enzymes, including α-amylase (AMY), β-amylase (BAM), isoamylase (ISA), and others.

Among starch-degrading enzymes, AMY (1,4-α-d-glucan-4-glucanohydrolase, E.C.3.2.1.1), which belongs to family 13 of glycosyl hydrolases (GH13 or GH13_6 in plants), has endoglycolytic activity and determines the first step in the starch breakdown pathway: hydrolysis of internal α-1,4-glycosidic linkages in granulated polymers. The released soluble glucans serve as substrates for other enzymes and further degradation [2].

Plant genomes may contain several AMY-encoding genes which may differ in their tissue and organ specificity. For example, the genome of *Arabidopsis thaliana* L. has three *AMY* genes encoding AtAMY1 (At4g25000) with a putative signal peptide, AtAMY2 (At1g76130) without any targeting signals, and AtAMY3 (At1g69830) with transit functions in chloroplasts [2]. Interestingly, the AtAMY1-AtAMY3 enzymes do not affect transitory starch degradation as their knockout mutants, alone or in combination, have no influence on starch hydrolysis in the leaves [3]. The lack of the effect may be due to the substitutive activity of other enzymes with similar functions, such as hydrolases (α-galactosidase (GH57), cyclomaltodextrinase (GH13, GH57), pullulanase (GH13)), transferases (amylosucrase (GH13), glucan-branching enzyme (GH13, GH57), alternansucrase (GH70)), isomerases (GH13), and HAT proteins (GH13) [4]. It is believed that AMYs are mainly involved in the breakdown of starch storage granules such as those in the seeds. Thus, intensive de novo synthesis of α-amylase has been observed during germination of cereal seeds [2], and the overexpression of the *TaAMY2* gene in wheat (*Triticum aestivum* L.) has led to an increase in the level of soluble carbohydrates (sucrose and α-gluco-oligosaccharide) in grain [5]. However, α-amylases may not be required at the initial stage of grain germination [5].

Several studies suggest that AMYs may be involved in the regulation of diverse physiologic processes in plants. Thus, it has been shown that in *A. thaliana*, leaves may secrete AtAMY1 during senescence or after biotic (*Pseudomonas syringae*) and abiotic (heat) stresses or in response to hormones such as gibberellins (GAs) and abscisic acid (ABA) [6]. A mutation in the *A. thaliana AMY1* gene activates flowering-time genes *CO* and *FT*, resulting in early flowering of the plant [7]. In apple (*Malus domestica* Borkh), the *MdAMY8* gene (homolog of AtAMY2), which is expressed at low levels in fruit, is significantly upregulated after cold exposure [8]. It has also been shown that in cereals, hormone-induced changes in the *AMY* expression accompany grain development and germination [9] and that in rice, the induction of the *AMY* genes and decrease in the ABA level are associated with the chalky grain phenotype [10].

The *AMY* gene families have been characterized in many plants. Thus, in potato (*Solanum tuberosum* L.), the *AMY* family comprises 20 genes [11], in cassava (*Manihot esculenta* Crantz), 6 genes [12], and in tea tree (*Camellia sinensis* (L.) Kuntze), 3 genes [13]. Among monocots, *AMY* families with various numbers of genes have been reported in banana (*Musa acuminate* L.; 12 genes) [14], wheat (*T. aestivum*; 6, 10, and 8 *AMY* genes in subgenomes A, B, and D, respectively) [9], rice (*Oryza sativa* L.; 10 genes) [15], and barley (*Hordeum vulgare* L.; 12 genes) [9]. In cereal, the *AMY* family is divided into subfamilies based on the protein pI value [9,15] or the presence of two *AMY* signatures [15,16].

A comprehensive phylogenetic study has classified the plant enzymes within the AMY family into six subfamilies, AMY1–AMY6. Among them, AMY1 and AMY2 are specific to monocots, AMY6 is found in basal land plants, basal angiosperms, two monocots, and major dicots (except for Brassicaceae and Rutaceae), and AMY3–AMY5 can be found throughout the entire green plant lineage, in monocots as well as dicots, and correspond to the three *A. thaliana AMYs*, AtAMY1–AtAMY3, respectively [17].

A dicot plant soybean (*Glycine max* (L.) Merrill) is included, along with major cereals, in the group of staple crops [18]. Soybean is the most important legume, oilseed, and protein crop, providing a source of beneficial macronutrients, antioxidants and phytochemicals [19,20]. Starch in mature soybean seeds accounts for about 1% of the seed dry weight, but its amount and the activity of the hydrolytic enzyme α-amylase can correlate with the protein and oil content, which is important in soybean breeding [21].

Another major aspect of soybean breeding is obtaining stress-resistant varieties because *G. max* plants are sensitive to various biotic and abiotic stresses, including infection, drought, salinity, and extreme temperatures [22]. The convergence point of many adverse effects is osmotic stress caused by water imbalance, which could be resolved by stomatal closure/opening under the control of ABA [23] followed by an increase in the concentration of soluble sugars in cells due to the breakdown of starch [24]. The involvement of ABA signaling in the response to abiotic stresses has also been demonstrated for soybean [25,26]. During seed development, elevated ABA levels may lead to the inhibition of AMY [9]. In leaves, the response to osmotic stress is accompanied by a simultaneous increase in ABA and the expression of *AMY3* and β-amylase *BAM1*, which synergistically degrade starch and promote the release of soluble sugars which is known to be involved in the response to osmotic stress [27].

Overall, these previous studies indicate that in soybean, the *AMY* genes are potentially important for the breeding of plant varieties with increased stress resistance and higher protein content in grain. However, information on the *AMY* family in *G. max* and its response to abiotic stresses is limited to some data on the AtAMY3 subfamily gene and changes in α-amylase activity in response to stress factors. It has been shown that the exposure of germinating soybean seeds and seedlings to salt stress alters starch accumulation and upregulates *GmAMY3* expression [28]. In soybean shoots and roots, drought affects starch content and release of soluble sugars and leads to an increase in α-amylase activity and *GmAMY3* expression [29]. Salt stress reduces starch content and increases α-amylase activity in soybean leaves and roots [30], but suppresses α-amylase activity in soybean seedlings [31]. In soybean seeds, drought stimulates α-amylase activity and accumulation of soluble sugars [32]. The use of zinc oxide nanoparticles under PEG-induced severe drought conditions has a positive effect on α-amylase activity in soybean seedlings and seeds [33]. It should be mentioned that soybean AMYs retain activity after treatment with heat and chemical denaturants (GndHCl), which is important given the use of α-amylases in industries (textile, food, pharmaceutical, biofuels, etc.) where the enzymes operate under harsh conditions [34].

The aim of this study was to identify and characterize genes of the α-amylase family in the *G. max* genome. We found five *AMY* genes belonging to the AtAMY1 (*GmaAMY5*), AtAMY2 (*GmaAMY4*), AtAMY3 (*GmaAMY1*, *GmaAMY3*), and AMY6 (*GmaAMY2*) subfamilies, and described them in terms of structure, phylogeny, chromosomal location, tissue expression patterns, and abiotic stress responses. The results should be useful for further investigation of the physiological role of α-amylases in soybean development and can be helpful in breeding experiments to obtain stress-resistant accessions.

## 2. Results

### 2.1. AMY Gene Family in G. max

Five full-length *AMY* genes (*GmaAMY1–GmaAMY5*) were detected in the *G. max* genome (Phytozome, Glycine_max_Wm82.a6.v1; NCBI, Glycine_max_v4.0); the numbering of the genes was based on the order of their location in the chromosomes. The *GmaAMY1–GmaAMY5* genes contained four or 12–14 exons (Table 1).

In addition to the *GmaAMY1–GmaAMY5* genes encoding proteins with the full-length catalytic part of the PLN02784 domain, three more genes homologous to *AtAMY1–AtAMY3* were identified, the products of which are annotated as α-amylases (Phytozome; the latest genome assembly, Wm82.a6.v1). These sequences encode truncated peptides (Glyma.17G261366, 141 aa; Glyma.11G153301, 101 aa; and Glyma.15G016000, 102 aa) corresponding to distinct segments of the PLN02784 catalytic domain (Supplementary Figure S1) and are likely the result of genome misassembly, incorrect gene annotation, or non-functionalization of duplicated copies following whole-genome duplication (WGD). Therefore, these three genes were not used in further analysis.

**Figure 1:**
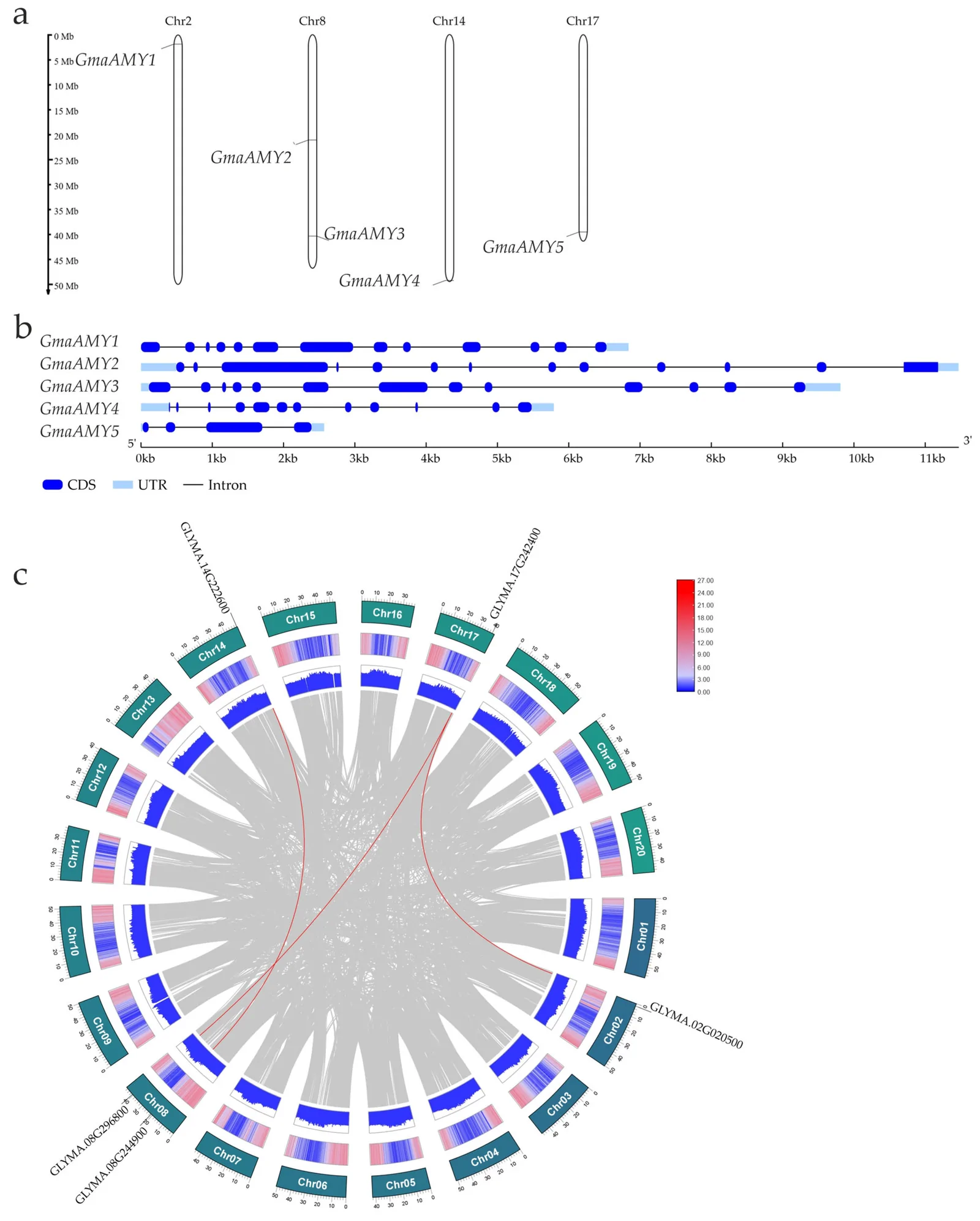
Chromosomal localization (**a**), exon–intron structure (**b**), and collinear relationships (**c**) of the *G. max AMY* genes.

*GmaAMY1*, *GmaAMY4*, and *GmaAMY5* were located on chromosomes 2, 14, and 17, respectively, and *GmaAMY2* and *GmaAMY3* were identified on the same chromosome 8 but at a significant distance from each other (Figure 1a). Interestingly, the exon–intron structures of the *GmaAMY2* and *GmaAMY3* genes were different, whereas those of *GmaAMY3* and *GmaAMY1* were similar, suggesting that the latter two genes, although located on separate chromosomes, may have been duplicated from a single ancestor (Figure 1b,c).

During evolution, soybean underwent two WGDs, and nearly 75% of the genes in the current paleopolyploid *G. max* genome are present in multiple paralogous copies [35]. To understand the duplication history of *AMY* genes in soybean, we analyzed their collinear relationships. Three collinear gene pairs (*GmaAMY5*/*GmaAMY1*, *GmaAMY5*/*GmaAMY3*, and *GmaAMY4*/*GmaAMY2*) were identified (Figure 1c), suggesting that at least four of the five *GmaAMY* genes may have originated through WGD.

**Figure 2:**
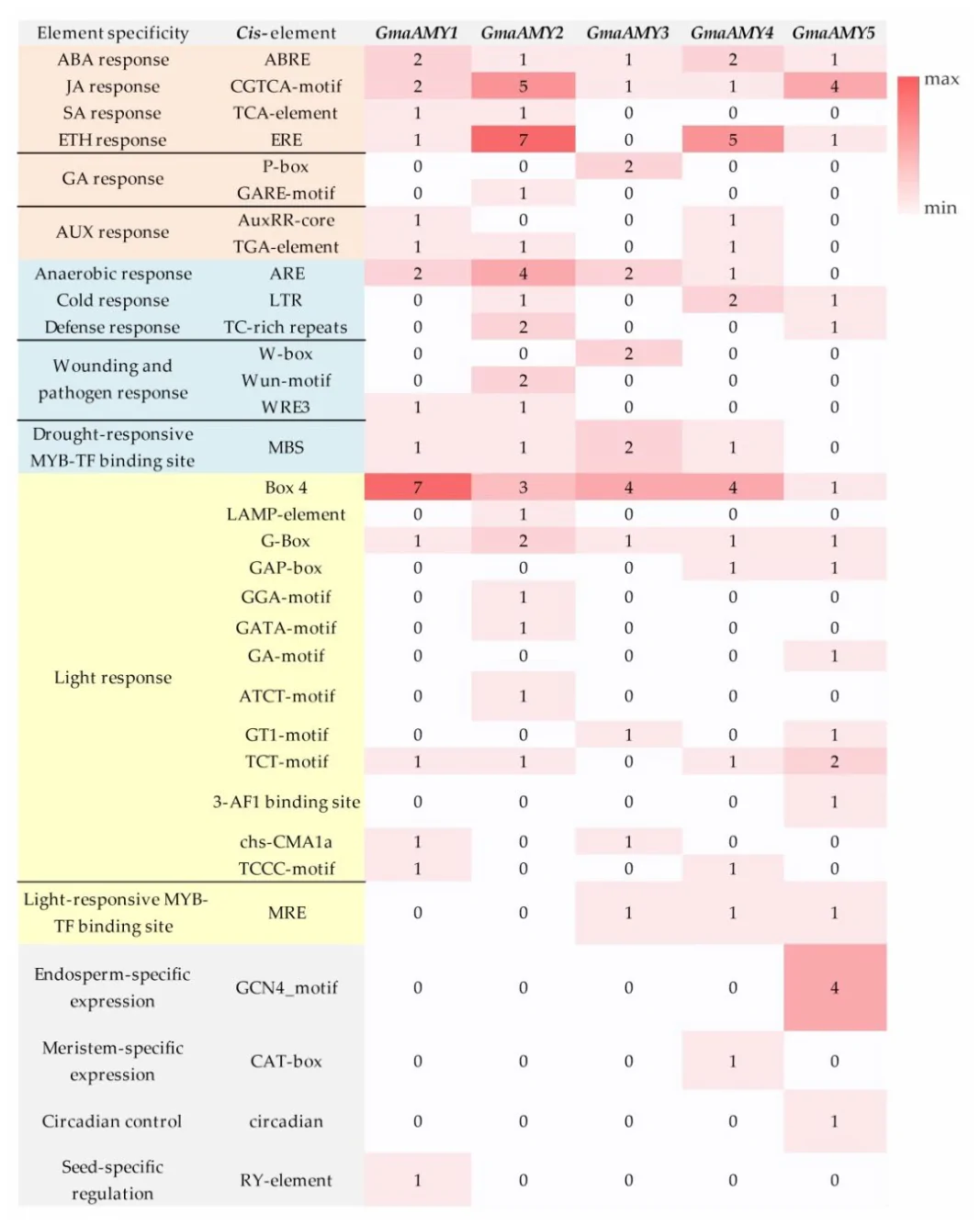
*Cis*-regulatory elements found in the *GmaAMY* promoters (2 kb) using PlantCARE. Color intensity (pale to dark) corresponds to the number of cis-elements (low to high). The different colors of the leftmost column divide the cis-elements into groups of hormone-sensitive (light orange), stress-sensitive (blue), light-sensitive (yellow), and other (pink).

**Figure 3:**
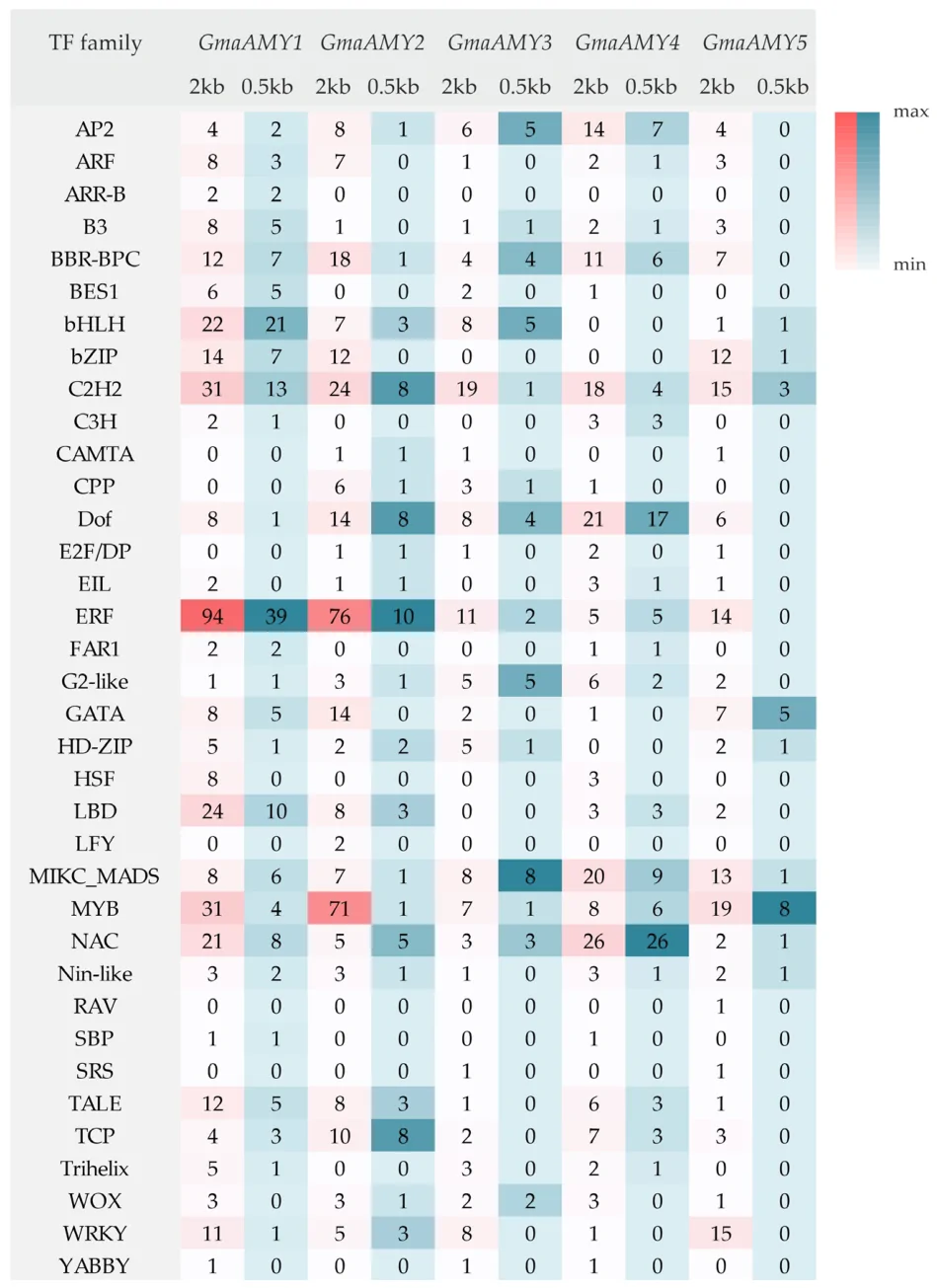
TF-binding sites found in the *GmaAMY* gene promoters. Analysis was performed using PlantTFDB. Promoter regions of 2 and 0.5 kb before the start codon are indicated in red and blue, respectively; color intensity (pale to dark) corresponds to the number of *cis*-elements (low to high).

To investigate potential transcriptional regulation of the *GmaAMY1–GmaAMY5* genes, we searched for *cis*-regulatory elements in their promoter/5′-UTR sequences (hereinafter referred to as promoter; Supplementary Table S1) using PlantCARE [36]. It was found that the 2 kb 5′-region before the start codon included important sites required for gene expression regulation in response to hormones, light, and stress factors, as well as those associated with site-specific gene expression in the meristem, seeds, and endosperm (Figure 2 and Figure 3).

The highest number of hormone-responsive elements was found in the promoters of *GmaAMY2* (response to methyl jasmonate (JA) and ethylene (ETH)), *GmaAMY4* (ETH), and *GmaAMY5* (JA), suggesting active involvement of these genes in the control of soybean development and adaptation. Salicylic acid (SA)-responsive elements were present in *GmaAMY1* and *GmaAMY2*, GA-responsive sites—in *GmaAMY2* and *GmaAMY3*, and auxin (AUX)-sensitive elements—and in *GmaAMY1*, *GmaAMY2*, and *GmaAMY4*. The smallest number of hormone-responsive elements was found in *GmaAMY3* (Figure 2). None of the genes contained sites associated with response to brassinosteroids, cytokinins, or strigolactones.

The *GmaAMY2* promoter had the highest total number of stress-responsive elements; however, *GmaAMY3* and *GmaAMY4* contained more drought- and cold-responsive sites, respectively (Figure 2).

All the *GmaAMY1–GmaAMY5* genes had sets of light-sensitive sites containing from 8 (*GmaAMY3*) to 11 (*GmaAMY1*) elements; among them, Box-4 was highly represented in most genes (*GmaAMY1–GmaAMY4*). However, only *GmaAMY5* contained a circadian-sensitive motif. Based on the sets of the other type elements found (Figure 2), it can be suggested that *GmaAMY5* should be specifically regulated in the endosperm, *GmaAMY4* in the meristem, and *GmaAMY1* in the seeds.

*Cis*-regulatory elements in gene promoters are mostly binding sites for gene transcription factors (TF). Next, we performed an in-depth search for TF-binding sites in the *GmaAMY1–GmaAMY5* promoters (2 and 0.5 kb variants) using the PlantTFDB database [37]. As a result, sites for 36 TF families were identified; among them, the most abundant were ERF, MYB, NAC, C2H2, and bHLH. Overall, the promoters of *GmaAMY1* and *GmaAMY2* were the most enriched in TF-binding sites, especially in those related to the ERF, bHLH, and C2H2 families. In the 2 kb vs. 0.5 kb promoter regions, *GmaAMY1* and *GmaAMY2* were abundant in the binding sites for MYB TFs, and *GmaAMY1* was also abundant in those for NAC TFs. In contrast, *GmaAMY4* had NAC-sites (26) primarily in the 0.5 kb promoter region (Figure 3). It should be noted that the 0.5 kb region of *GmaAMY4* was also enriched with elements for Dof (17) and MIKC_MADS (9), whereas that of *GmaAMY3* was enriched with sites for MIKC_MADS (8), G2-like (5), bHLH (5), and AP2 (5) TF families. The *GmaAMY5* promoter appeared to be the least enriched in TF elements, but it still had well-represented sites for MYB and GATA families in both 2 kb and 0.5 kb regions (19/8 and 7/5, respectively) (Figure 3).

*GmaAMY* genes also contain unique binding sites for TFs ARR-B (*GmaAMY1*), HSF (*GmaAMY4*), LFY (*GmaAMY2*), RAV (*GmaAMY5*), SRS (*GmaAMY3* and *5*), and SBP and FAR1 (*GmaAMY1* and *4*) (Figure 3).

In general, the promoters of all five *GmaAMY* genes were shown to contain binding sites for TFs of the AP2, ERF, NAC, MYB, WRKY, DOF, bZIP, and MIKC-MADS families (except for bZIP in *GmaAMY3* and *GmaAMY4*) (Figure 3), representatives of which are directly or indirectly involved in the regulation of starch metabolism genes, including the expression of *AMY* genes (the AP2 and DOF families) [38].

Most transcription factor families that can bind to *GmaAMY* promoters are involved in plant stress responses, and the presence of binding sites for them is consistent with the presence of stress- and hormone-responsive *cis*-elements. For example, the *GmaAMY2* gene promoter, which showed the second-highest (after *GmaAMY1*) number of TF-binding sites, also had the highest number of stress- and hormone-responsive elements (11 and 16 sites) (Figure 2 and Figure 3).

Hormone-responsive *cis*-element profiles do not always correlate with the binding sites for TFs involved in specific hormonal signaling. For example, the *GmaAMY3* promoter lacks ETH-responsive *cis*-elements (Figure 2) but does have binding sites for the ERF transcription factors (Figure 3). The promoters of all five *GmaAMY* genes are characterized by the presence of binding sites for the BES1 and ARR-B transcription factors, but do not contain brassinosteroid- and cytokinin-responsive *cis*-elements (Figure 2).

To assess potential post-transcriptional regulation of *GmaAMY* genes, we performed a prediction analysis of miRNA target sites in the mRNA sequences using PsRNATarget [39] and found 16 (*GmaAMY1*), 12 (*GmaAMY2*), 10 (*GmaAMY3*), 4 (*GmaAMY4*), and 5 (*GmaAMY5*) sites for various miRNAs. Each gene had a unique set of miRNA-specific sites, with the exception of 5 sites targeted by the same miRNAs in *GmaAMY1* and *GmaAMY2* (*gma-miR1513a-3p* and *gma-miR2119*) and *GmaAMY2* and *GmaAMY3* (*gma-miR1535a*, *gma-miR1535b*, and *gma-miR9742*) (Supplementary Table S2). Among the predicted miRNAs potentially targeting *GmaAMY1–GmaAMY5* mRNAs, most have been shown to be associated with plant stress responses [40,41,42,43,44]; 13 miRNAs are involved only in the regulation of organ development [45,46,47,48] or nitrogen fixation in nodules [49].

### 2.2. Putative GmaAMY Proteins

Putative *GmaAMY1–GmaAMY5*-encoded protein products can be divided into two groups in terms of size: GmaAMY1–GmaAMY3 (922–957 aa) and GmaAMY4 and GmaAMY5 (413–414 aa) (Supplementary Table S1). Based on MW, pI, AI, II, and GRAVY values (Table 2), the proteins were predicted to be hydrophilic and globular; GmaAMY1–GmaAMY3 and 5 were acidic and GmaAMY4 was alkaline; GmaAMY3 and GmaAMY5 were stable, whereas GmaAMY1, GmaAMY2, and GmaAMY4 were unstable.

Subcellular localization analysis (WoLF PSORT) revealed that GmaAMY1–GmaAMY4 could be localized in the chloroplasts or nucleus, whereas GmaAMY5 could be present in various cellular compartments, including chloroplasts, nucleus, and vacuoles, and could also be secreted into the extracellular space, which is consistent with the prediction of an N-terminal SP (Table 2).

**Figure 4:**
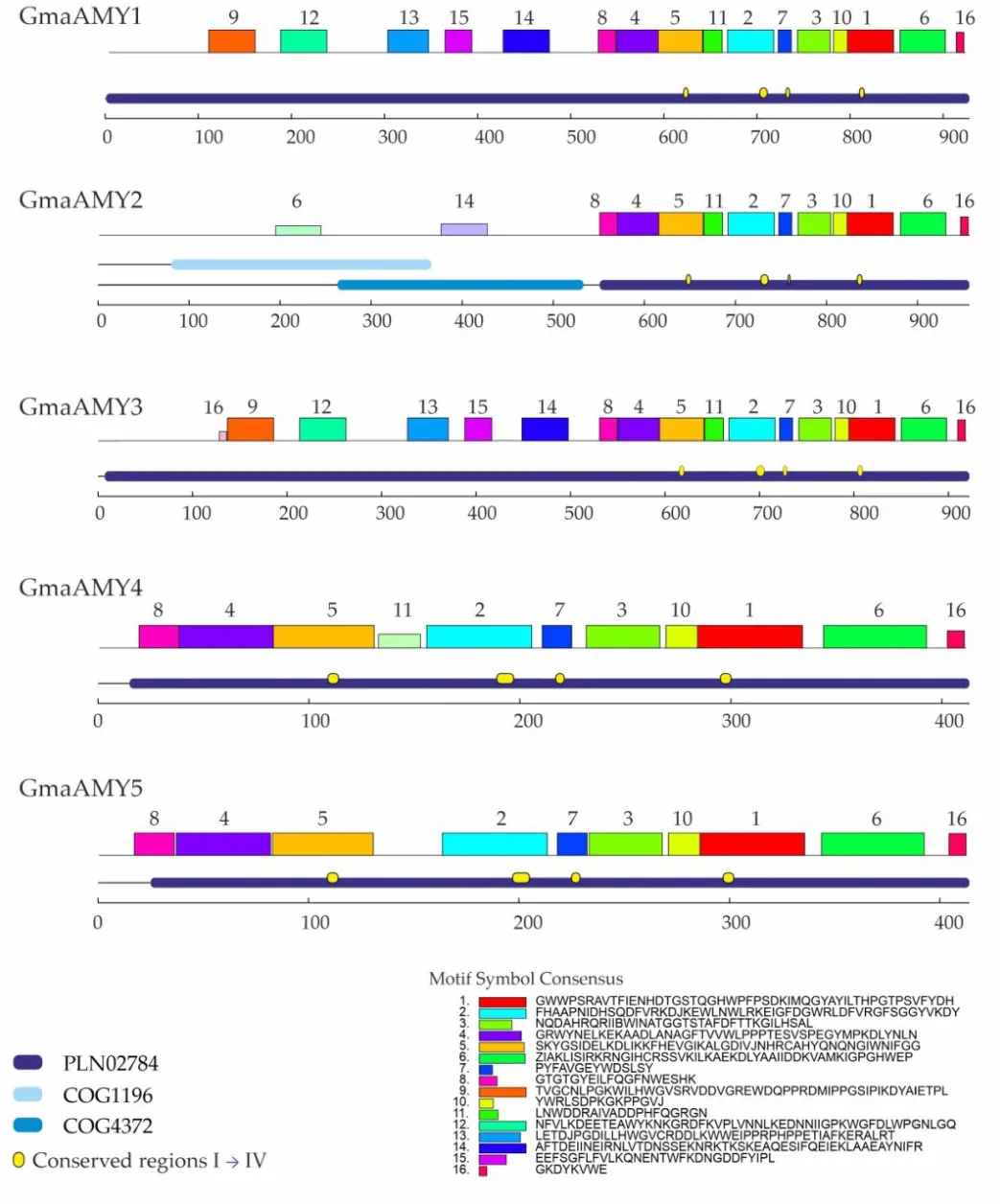
Distribution of conserved domains and motifs in GmaAMY1–GmaAMY5 protein sequences.

All GmaAMY1–GmaAMY5 proteins contained conserved α-amylase domain PLN02784, although GmaAMY2 carried its truncated version; the latter protein also had two other domains (COG1196 and COG4372) (Table 2, Figure 4), which may be a result of gene misassembly. All five proteins had four highly conserved α-amylase signatures I–IV defined according to [50] and enzyme active sites (according to NCBI-CDD and [34]) (Table 2, Figure 4).

Prediction analysis of conserved motifs (MEME 5.5.9) suggested the presence of 16 consensus sequences in GmaAMY1–GmaAMY5; among them, 11 constituted the C-terminal region of the PLN02784 domain (except for motif 11 missing in GmaAMY5), which included all found catalytic sites and α-amylase signatures (Table 2, Figure 4). The catalytic activity of this region was indirectly confirmed by building 3D models of GmaAMY1–GmaAMY5 (Phyre2.2) based on closely matched crystal structures of two α-amylase homologs from barley: isozyme 1 (PDB: 1HT6; entry DOI: 10.2210/pdb1ht6/pdb [52]), 48% identical to GmaAMY1–GmaAMY4 and 62% identical to GmaAMY5, and isozyme 2 (PDB: 1AVA; entry DOI: 10.2210/pdb1ava/pdb [53]), 50% identical to GmaAMY1 and GmaAMY4, 49% to GmaAMY2 and GmaAMY3, and 67% to GmaAMY5. On average, 41–42% of the GmaAMY1–GmaAMY3 and 93–94% of the GmaAMY4 and GmaAMY5 sequences were modeled with 100% confidence; the reliably constructed parts of GmaAMY1–GmaAMY3 corresponded to the C-terminal region of the PLN02784 domain.

Overall, a high-confidence 3D structure was predicted only for the C-terminal portion of the PLN02784 domain of GmaAMY1–GmaAMY5 proteins (Supplementary Figure S2). This region represents the catalytic domain A of α-amylases. It consists of eight β-strands interspersed with eight α-helices. Between β-strand 3 and α-helix 3 lies the B domain, which determines the binding of calcium ions, the formation of the substrate-binding cleft and the substrate specificity of the enzyme; the C-terminal β-strands of A-domain form part of the starch granule-binding domain [54]. The catalytic domain A of GmaAMY1–GmaAMY5 proteins was predicted to have the above-mentioned stable (β/α)8-barrel topology and varied in position: GmaAMY1 (position 536–927 aa), GmaAMY2 (564–957 aa), GmaAMY3 (532–922 aa), GmaAMY4 (24–414 aa), and GmaAMY5 (26–413 aa) (Supplementary Figure S2). Domain B was localized in the GmaAMY1–GmaAMY5 sequences at positions 624–682, 649–706, 617–675, 113–167 and 111–175 aa, respectively. The C-terminal portion of the A-domain, which is involved in starch granule binding, was represented by five β-strands in all five proteins. In domains B and A of GmaAMYs, two calcium binding sites were consistently present (Table 2), which may ensure the creation and stabilization of the active center [34]. The GmaAMY1–GmaAMY5 proteins were shown to contain an Asp residue (at positions 707, 732, 701, 193, and 201, respectively, Table 2), similar to the Asp666 in AtAMY3, the mutation of which abolishes enzyme activity [55]. In GmaAMY1 and GmaAMY3, residues Cys540/Cys628 and Cys534/Cys622, respectively, which corresponded to residues Cys499/Cys587 in AtAMY3, were identified (Table 2), which are shown to be important for catalytic activity and to play a central role in the redox regulation of the AtAMY3 enzyme [55]; GmaAMY2 had only one of such residues, Cys653.

Motifs 9 and 12–15 corresponded to the N-terminal part of the PLN02784 domain, which did not contain active sites or signatures required for α-amylase function; it was present in GmaAMY1 and GmaAMY3 and absent in GmaAMY2, GmaAMY4 and GmaAMY5 (Table 2, Figure 4). Alignment of GmaAMY1 and GmaAMY3 with their closest homolog AtAMY3 revealed the presence of two starch-binding domains from the carbohydrate-binding module 45 (CBM45) family: CBM45-1/CBM45-2 at position 98–214/290–397 aa in GmaAMY1 and 101–227/306–410 aa in GmaAMY3 (Supplementary Figure S3). These domains may mediate the interaction with the starch granule surface, as shown for AtAMY3 [55,56].

Phylogenetic analysis of soybean α-amylases included protein sequences typical for each of the AMY1–AMY6 subfamilies, according to [17]. As is known, α-amylases of dicotyledonous plants (including soybean) belong to the AMY3–AMY6 subfamilies; AMY3-AMY5 correspond to AtAMY1–AtAMY3 of *A. thaliana* (hereinafter, the abbreviation AtAMY1–AtAMY3 is used, as is customary for dicotyledonous species [12]), and AMY6 is not represented in *Arabidopsis* [17]. The analysis confirmed the evolutionary similarity of GmaAMY proteins with their homologues from dicotyledonous plants and their divergence from α-amylases of monocotyledons such as *O. sativa* and *H. vulgare*, which is consistent with previous studies [17].

**Figure 5:**
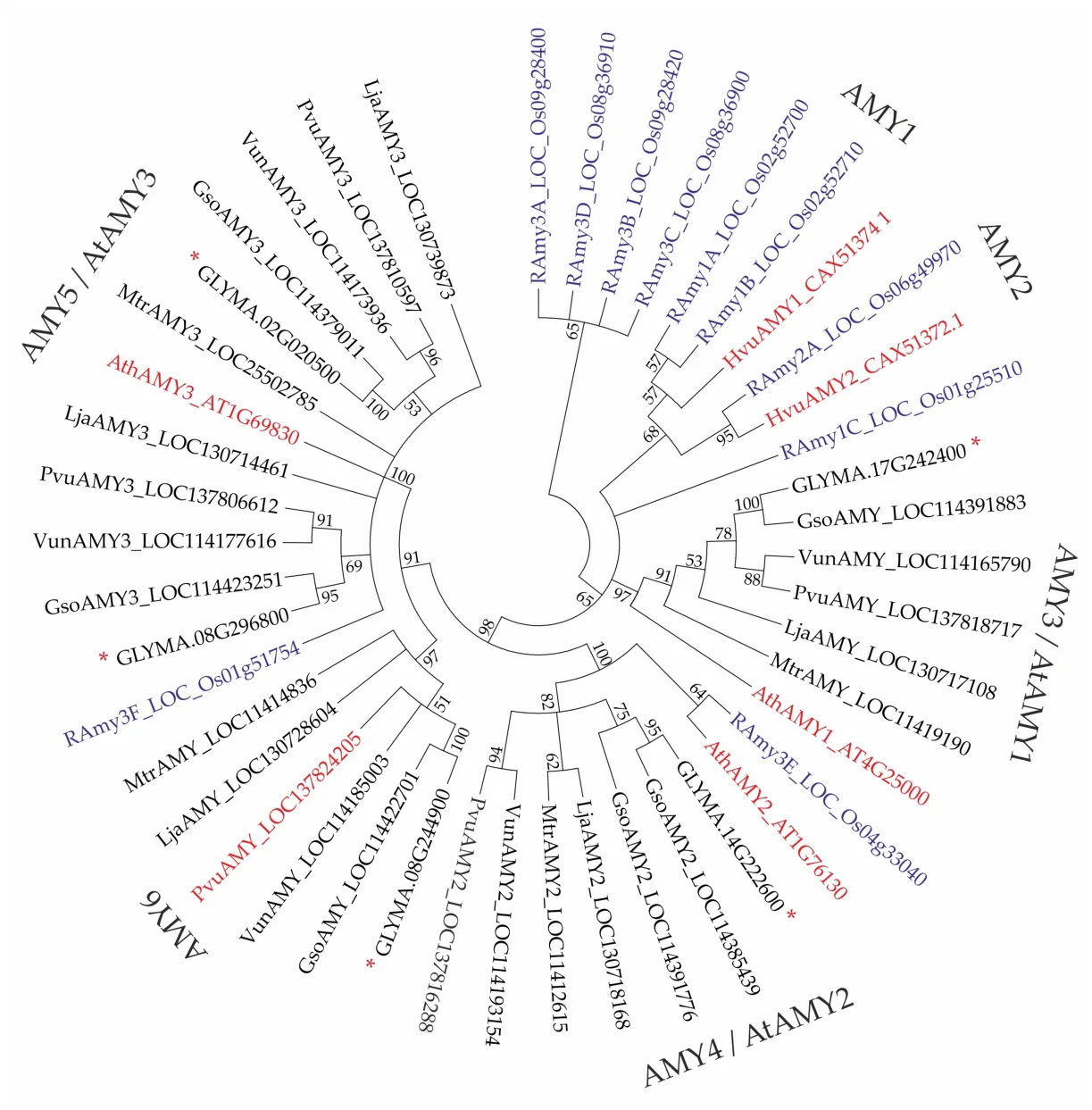
Evolutionary relationships between α-amylases of *G. max* (Gma, red stars), *A. thaliana* (Ath), *O. sativa* (Osa, blue), *H. vulgare* (Hvu), and *Glycine soja* (Gso), *Phaseolus vulgaris* (Pvu), *Vigna unguiculata* (Vun), *Medicago truncatula* (Mtr), and *Lotus japonicas* (Lja) (all black). The names of proteins typical of subfamilies AMY1–AMY6 (according to [17]) are highlighted in red: HvuAMY_CAX51374_1 (AMY1 subfamily), HvuAMY_CAX51372.1 (AMY2), AthAMY1 (AMY3/AtAMY1), AthAMY2 (AMY4/AtAMY2), AthAMY3 (AMY5/AtAMY3), and PvuAMY_LOC137824205 (AMY6). The unrooted dendrogram was constructed in MEGA 7.0.26 (the Maximum Likelihood method based on the JTT model; bootstrap with 1000 replicates) [57].

GmaAMYs could be divided into subfamilies AtAMY1 (GmaAMY5), AtAMY2 (GmaAMY4), AtAMY3 (GmaAMY1, GmaAMY3), and AMY6 (GmaAMY2). The subfamilies AtAMY3 and AMY6 are evolutionarily close and appear as two related clusters within one subfamily (Figure 5), as in the study [17].

*O. sativa* proteins formed distinct clusters, with the exception of OsaRAmy3E and OsaRAmy3F, which fell into subfamilies AtAMY2 and AtAMY3 of dicotyledonous plants, respectively (Figure 5). *OsaRAmy3E* is known to be expressed in the aleurone layer during germination and seed development, and *OsaRAmy3F* is expressed in all tissues throughout plant growth [15]; both are also involved in stress responses to cold, drought, and salinity (RGAP.r7 data). The remaining *O. sativa* proteins were either grouped remotely from the others (a cluster of RAmy3A, RAmy3B, RAmy3C, and RAmy3D) or were assigned to the monocotyledonous-specific (according to [17]) subfamilies AMY1 (RAmy2A) and AMY2 (RAmy1A/RAmy1B) (Figure 5). The distribution of rice proteins on the dendrogram corresponds to the study [15], with the exception of OsaRAmy1C, which forms a separate branch (Figure 5) instead of being included in the AtAMY1 subfamily (as in study [15]).

Thus, GmaAMY1–GmaAMY5 were predicted to be hydrophilic globular proteins with a predominantly cytoplasmic localization (except GmaAMY5, which has an SP and is likely secreted). All GmaAMY1–GmaAMY5 contained the highly conserved PLN02784 domain, α-amylase signatures, and active sites, indicating functional similarity among the proteins. The presence of an N-terminal extension in GmaAMY1–GmaAMY3 and CBM45-1/2 modules in GmaAMY1 and GmaAMY3, as well as phylogenetic clustering separately from GmaAMY4 and GmaAMY5, may determine functional differences between GmaAMY1/GmaAMY3, GmaAMY2 and GmaAMY4/GmaAMY5.

### 2.3. Organ-Specific Expression Patterns of the GmaAMY1–GmaAMY5 Genes

**Figure 6:**
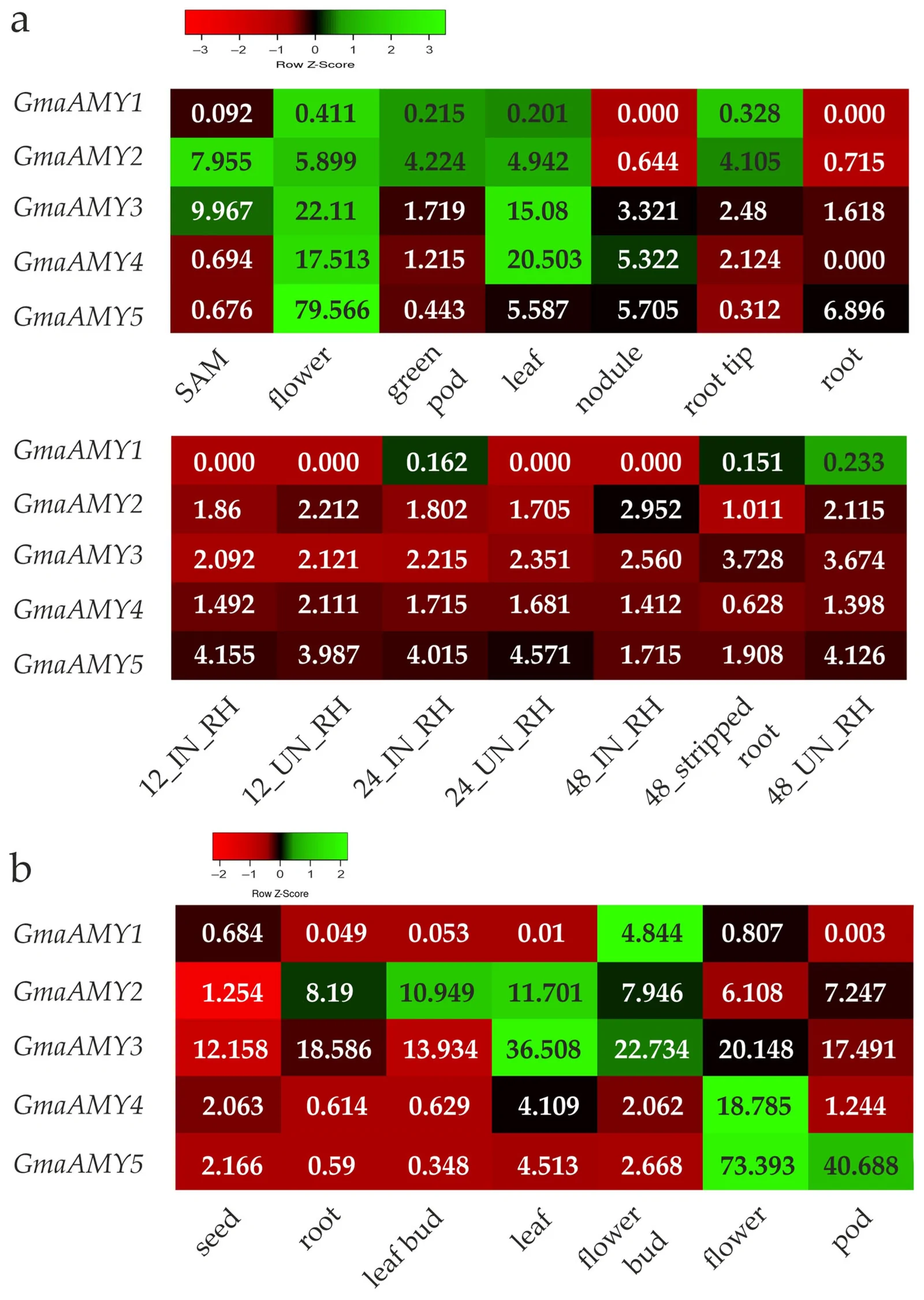
Heatmap of α-amylase mRNA levels in *G. max* organs based on normalized RNAseq data in TPM (transcripts per million) format. (**a**) (top) expression in SAM, flower, green pod, leaf, root tip, and root; (bottom) expression in root hairs after 12, 24, and 48 h of pathogen (*Bradyrhizobium japonicum*) infection (12_,24_,48_IN_RH) compared to uninfected control (12_,24_,48_UN_RH), and stripped roots at 48 h according to the *G. max* integrated transcriptome atlas [58,59]. (**b**) Expression in seeds, roots, leaf buds, leaves, flower buds, flowers, and pods according to SoyTFBase.

Gene expression was analyzed based on the transcriptome data available in the GERS, LIS (Figure 6a), and SoyTFBase (Figure 6b) resources.

According to GERS and LIS data, all *GmaAMY1–GmaAMY5* genes were highly expressed in the flowers but very weakly in the roots; low expression levels were detected for *GmaAMY1–GmaAMY3* and *GmaAMY5* in mature nodules, for *GmaAMY3–GmaAMY5* in green pods, for *GmaAMY1*, *GmaAMY4*, and *GmaAMY5* in apical meristem, and for *GmaAMY5* in the leaves (Figure 6a). In root hairs, the expression levels of *GmaAMY1*, *GmaAMY2*, and *GmaAMY5* changed in response to pathogen infection, with a significant increase in expression of the *GmaAMY1* gene 48 h after infection (Figure 6a).

According to SoyTFBase data, relatively high expression levels of some *GmaAMY* genes were observed in individual organs: seeds (*GmaAMY1*), roots and leaf buds (*GmaAMY2*), leaves (*GmaAMY2–GmaAMY4*), flower buds (*GmaAMY1–GmaAMY3*), flowers (all except *GmaAMY2*), and siliques (*GmaAMY2*, *GmaAMY5*) (Figure 6b).

Inconsistency in gene expression patterns across databases (e.g., high (Figure 6a) or low (Figure 6b) expression of *GmaAMY1–GmaAMY3* in the flowers and *GmaAMY1* and *GmaAMY5* in the leaves) may be due to the generation and processing of different transcriptomes, as well as to variations between sequenced soybean genotypes, developmental stages, growing conditions, sampling methods, and sequencing platforms.

### 2.4. Expression of GmaAMY1–GmaAMY5 and Carbohydrate Content in G. max Leaves in Response to Salinity, PEG-Induced Osmotic Stress, and Cold

We analyzed the *GmaAMY1–GmaAMY5* expression dynamics in soybean cv. Doka leaves in response to abiotic stresses.

The real-time PCR results revealed weak expression of the *GmaAMY1* gene, which was, depending on the organ, approximately 4–380 times lower than that of *GmaAMY3* and *GmaAMY4* and 2–59 times lower than that of *GmaAMY2* and *GmaAMY5* (Supplementary Figure S4).

**Figure 7:**
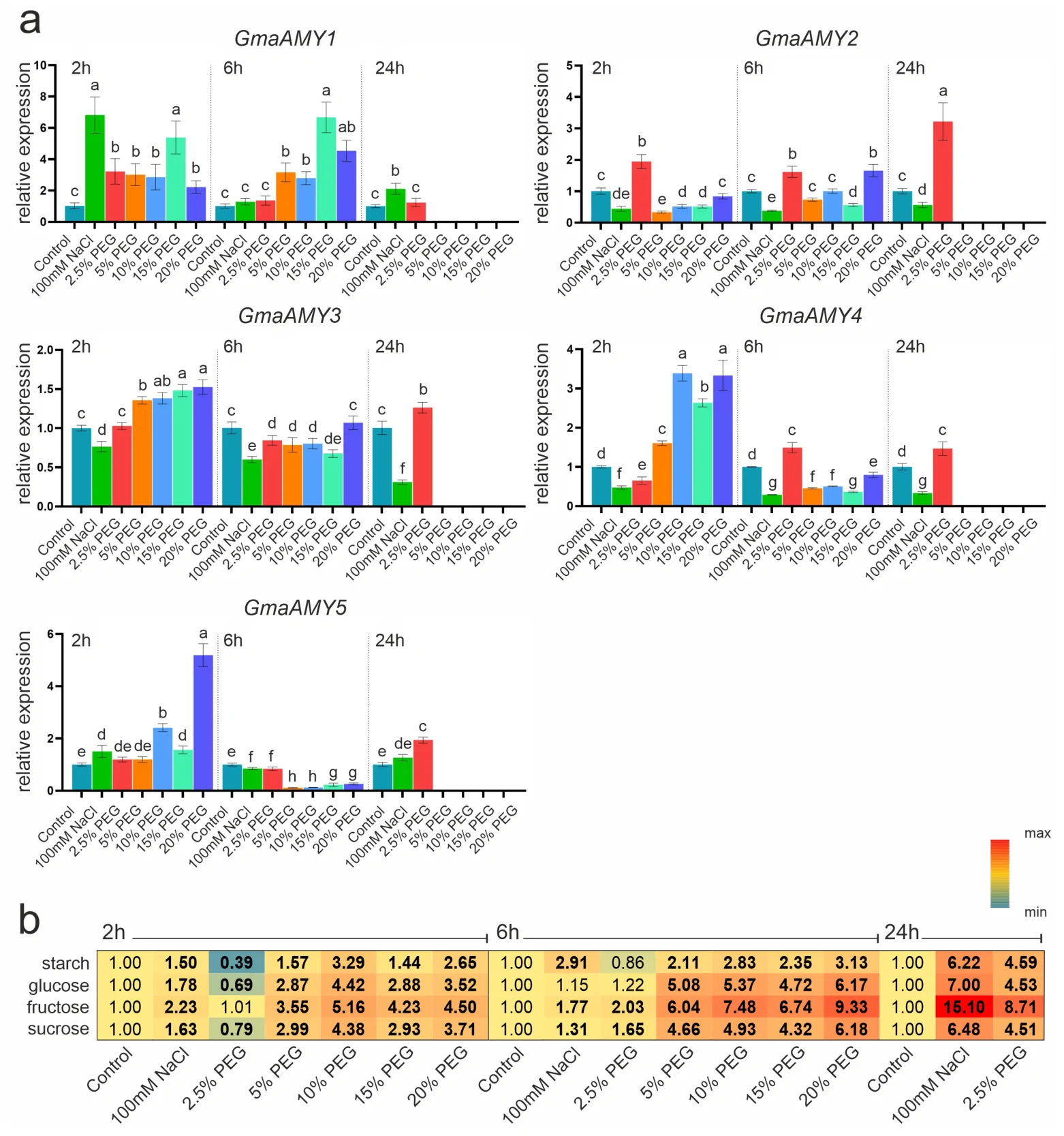
Changes in the expression levels of *GmaAMY1–GmaAMY5* genes (**a**) and carbohydrate content (**b**) in the leaves of soybean cv. Doka in response to stresses. *G. max* seedlings were exposed to salinity (100 mM NaCl) and PEG (2.5, 5, 10, 15, and 20%) for 2, 6, and 24 h and analyzed for *GmaAMY1–GmaAMY5* transcription and carbohydrate content compared to untreated control plants. As 5–20% PEG caused plant death after 24 h exposure, gene expression and starch content were analyzed only at 2.5% PEG. (**a**) Expression levels are presented relative to control (taken as 1). The data are shown as the mean ± SD (*n* = 9); ^a–h^, *p* < 0.01 indicates significant differences between all samples. (**b**) Heatmap of starch, glucose, fructose, and sucrose content (mg/g) change in response to salinity and PEG-induced stress. The color gradient and numbers (the ratio of the experimental value to the control value) in the cells show the level and direction of change in carbohydrate content in a stressed plant. Values corresponding to statistically significant changes in content are highlighted in bold.

Under salinity stress, gene expression changes demonstrated two patterns. The first one was a dynamic three-phase expression pattern: *GmaAMY1* and *GmaAMY5* levels increased sharply after 2 h, decreased almost to control values after 6 h, and increased again after 24 h. The second pattern was observed for *GmaAMY2–GmaAMY4*, when the expression decreased after 2 h and was maintained at a reduced level or further decreased at later time points (Figure 7). The expression changes were accompanied by an increase in the amount of soluble sugars in the leaves, starting already after 2 h; the starch content was also increased but at a later point (6 h) (Figure 7, Supplementary Figure S5).

Under PEG-induced drought, plants survived for 24 h only at 2.5% PEG. At this PEG concentration, gene expression was upregulated (*GmaAMY1* and *GmaAMY2*), downregulated (*GmaAMY4*), or remained unchanged (*GmaAMY3* and *GmaAMY5*) after 2 h, and further increased *(GmaAMY2–GmaAMY5*) or decreased (*GmaAMY1*) after 24 h (Figure 7). Starch content started to decrease after 2 h of treatment with 2.5% PEG but then increased up to the control value (6 h) and above it (24 h). Soluble sugar content did not change at 2 h but increased significantly at 24 h (Figure 7).

Treatment of plants with 5–20% PEG for 2 h caused sharp upregulation of *GmaAMY1* and *GmaAMY3–GmaAMY5* and downregulation of *GmaAMY2*; the exception was unchanged expression of *GmaAMY5* at 5% PEG and *GmaAMY2* at 20% PEG. After 6 h of stress, *GmaAMY1* remained upregulated and *GmaAMY2 was* downregulated at 5 and 15% or upregulated at 10 and 20%, whereas *GmaAMY3–GmaAMY5* were downregulated (Figure 7). At all four PEG concentrations, the content of starch and soluble sugars was significantly increased both at 2 and 6 h (Figure 7).

**Figure 8:**
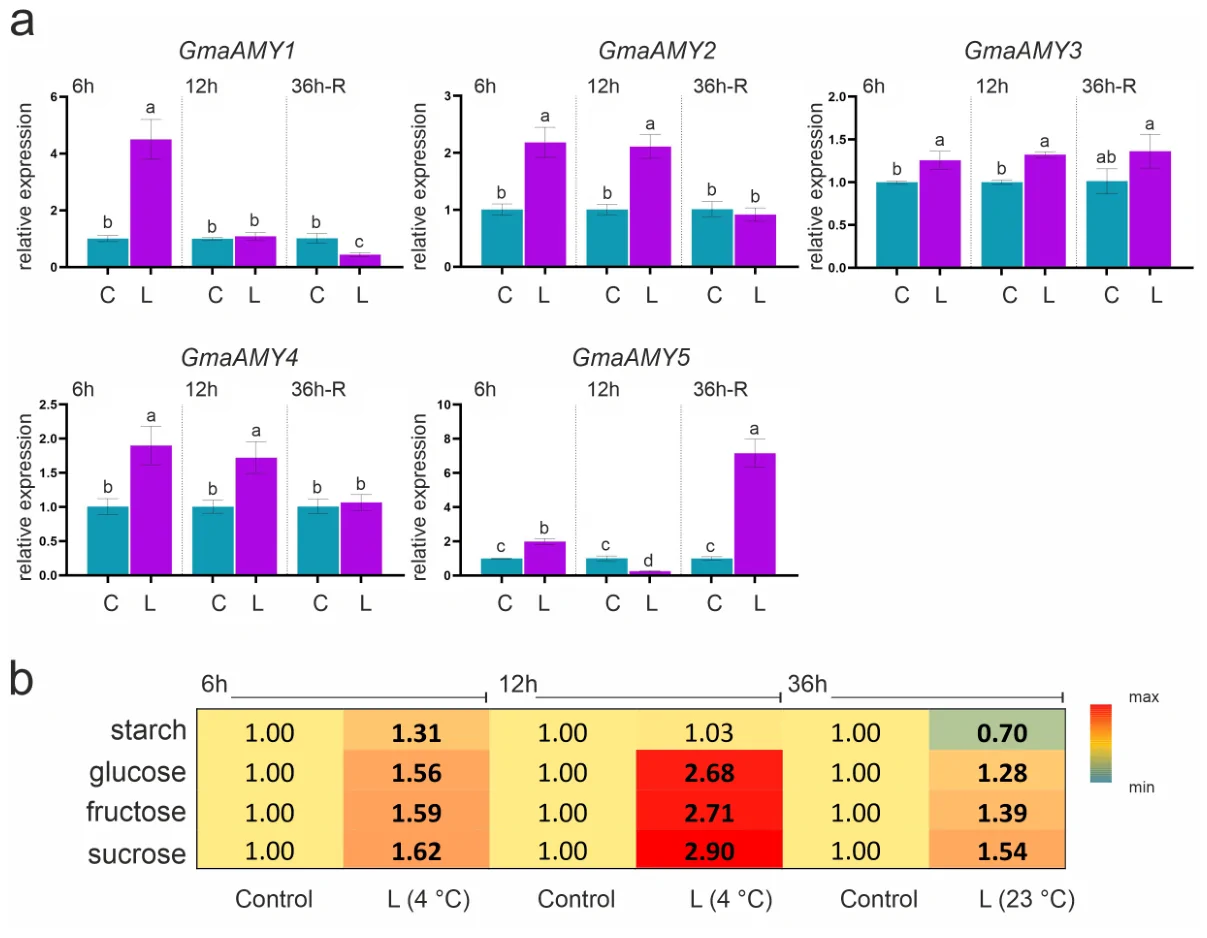
Changes in the expression levels of *GmaAMY1–GmaAMY5* genes (**a**) and carbohydrate content (**b**) in the leaves of soybean cv. Doka in response to low temperature (L). *G. max* seedlings were exposed to cold (4 °C) for 6 and 12 h and then were returned to the normal conditions for 36 h (recovery, 23 °C, 36 h-R). L, plants exposed to cold; C, untreated control plants. (**a**) Transcription levels are presented relative to control (taken as 1) and carbohydrate content—as absolute values. The data are shown as the mean ± SD (*n* = 9); ^a–d^, *p* < 0.01 indicates significant differences between all samples. (**b**) Heatmap of starch, glucose, fructose, and sucrose content (mg/g) change in response to 6–12 h cold stress and 36 h recovery. The color gradient and numbers (the ratio of the experimental value to the control value) in the cells show the level and direction of change in carbohydrate content in a stressed plant. Values corresponding to statistically significant changes in content are highlighted in bold.

After the first 6 h of cold exposure (4 °C), all five genes were significantly upregulated, but by 12 h only *GmaAMY2–GmaAMY4* retained increased transcript levels, whereas *GmaAMY1* and *GmaAMY5* transcripts were decreased to and below control, respectively. After 36 h of post-stress recovery, the expression of *GmaAMY2* and *GmaAMY4* was normalized, whereas that of *GmaAMY3* and *GmaAMY5* was upregulated and that of *GmaAMY1* downregulated relative to control (Figure 8). The starch content in the leaves was first increased (6 h), then decreased to the control level (12 h), and further decreased after recovery (36 h-R). The content of soluble sugars was significantly increased during cold stress (6–12 h) but decreased almost to the control level after the recovery period (Figure 8, Supplementary Figure S5).

Thus, various stress conditions affected both *GmaAMY1–GmaAMY5* gene expression and carbohydrate content. These data indicate that although starch degradation is regulated by multiple genes, *GmaAMY1–GmaAMY5* could play an important role in the process. Therefore, we analyzed possible links between starch hydrolysis and *GmaAMY1–GmaAMY5* expression during stress responses. The results indicated that under salinity stress, there was a significant inverse correlation between starch content and *GmaAMY4* expression (correlation coefficient (r) = −0.5135, *p*-value = 0.0293) and the overall transcription of all five genes taken together (r = −0.6318, *p* = 0.0049). The expression of the *GmaAMY2, GmaAMY3*, and *GmaAMY5* genes also tended to be associated with starch content (r = −0.3434, −0.4613, −0.2461, respectively) but not significantly (*p* > 0.05). Under PEG stress, inverse relationship with starch content was found for *GmaAMY5* (r = −0.2294, *p* = 0.3447), whereas under cold stress there was such correlation with *GmaAMY3* (r = −0.2289), albeit statistically insignificant (*p* = 0.5248).

Overall, the expression of all five genes in the leaves changed in response to salinity, PEG, and cold—factors that include osmotic stress. Given that adaptation to such stress is closely linked to the control of cellular carbohydrate metabolism, the observed differential expression of *GmaAMY1–GmaAMY5* (Figure 7 and Figure 8) suggests a role for α-amylases in the regulation of defense responses in soybean through starch breakdown. This notion is supported by the negative correlation found between the expression of individual *GmaAMY* genes and starch content; a low level of statistical significance for some *GmaAMY*s could be associated with the multigene nature of the starch degradation process, in which α-amylases are not the only participants; the ongoing synthesis of starch could also be a factor.

The high expression levels of GmaAMY3 and GmaAMY4 in the leaves may point to a considerable role of the encoded enzymes in chloroplast-specific starch degradation at this stage of soybean development; GmaAMY2 and GmaAMY5 with moderate expression may also participate in the process. At the same time, low expression of GmaAMY1 may indicate a minor contribution of the corresponding α-amylase to starch breakdown.

## 3. Discussion

Alpha-amylases are considered important enzymes influencing carbohydrate metabolism in plants during development and stress response [2]. In this study, we identified and characterized five genes, *GmaAMY1–GmaAMY5*, encoding putative α-amylases in soybean. According to the generally accepted division of dicotyledonous α-amylases into three subfamilies (based on *Arabidopsis* enzymes AMY1–AMY3) [2], we classified the five soybean genes into groups AtAMY1 (*GmaAMY5*), AtAMY2 (*GmaAMY4*), AtAMY3 (*GmaAMY1*–*GmaAMY3*) (Figure 5). According to the distribution of α-amylases of land plants into six subfamilies [17], we assigned GmaAMY2 to the AMY6 subfamily, which, given the results of phylogenetic analysis (Figure 5), is rather a divergent branch of the AtAMY3 subfamily.

The initial hypothesis about functions of soybean α-amylases was made based on the phylogeny and established properties of *A. thaliana* AMY enzymes [2,6,56]. Thus, GmaAMY5, similar to AtAMY1, has an SP and could be secreted to hydrolyze starch in the extracellular space. GmaAMY1 and GmaAMY3, similar to AtAMY3, may be chloroplast α-amylases regulated by redox processes, whereas GmaAMY4 may be involved in the response of soybean to cold stress, similar to the apple AtAMY2 homolog MdAMY8 [8].

The genes of subfamilies AtAMY1 (*GmaAMY5*) and AtAMY2 (*GmaAMY4*) are located on different chromosomes and have no similarity in the exon–intron structure either with each other or with the genes of the AtAMY3 subfamily (Figure 1). The *GmaAMY1* and *GmaAMY3* genes of subfamily AtAMY3 have close similarity in the exon–intron structure (Figure 1) as well as in *cis*-regulatory elements in promoters (Figure 2), suggesting their conserved function. The expression of both genes can be dependent on hormones (ABA, JA), light, wounding, and drought (Figure 2), which is consistent with the involvement of *Arabidopsis AMY3* in stress-induced starch degradation via ABA signaling [28]. Unlike *GmaAMY3*, *GmaAMY1* lacks GA-responsive sites but can be linked to SA, ETH, and AUX signaling pathways and is regulated specifically in seeds (Figure 2). The exon–intron structure of *GmaAMY2*, the member of the AMY6 subfamily (closely related to AtAMY3), differs significantly from those of *GmaAMY1* and *GmaAMY3* (Figure 1). Based on the *cis*-element composition, the role of *GmaAMY2* may be associated with the ABA, MeJA, SA, ETH, GA, and AUX signaling, light, drought, and cold response (Figure 2).

The profiles of *cis*-regulatory elements in the other *GmaAMY* genes indicate that soybean α-amylases may be involved in starch degradation associated with the signaling of phytohormones ABA, MeJA, ETH (*GmaAMY4*, *GmaAMY5*), ETH (*GmaAMY4*, *GmaAMY5*), and AUX (*GmaAMY4*), responses to light and cold (*GmaAMY4*, *GmaAMY5*), drought (*GmaAMY4*), and circadian rhythms (*GmaAMY5*). Regulation of the *GmaAMY4* and *GmaAMY5* genes may occur specifically in the meristem and endosperm, respectively (Figure 2).

Considering that the 5′-regulatory regions of all *GmaAMY* genes contain multiple binding sites for TFs of various families (Figure 3), most of which are critically involved in plant development and stress responses (for example, NAC [60], MYB [61], or WRKY [62]), it can be presumed that *GmaAMY1–GmaAMY5* have roles in the regulation of soybean development and adaptation.

Each of the five genes contains a distinct set of miRNA target sites (Supplementary Table S2). Analysis of the functional characteristics of these sites [40,41,42,43,44,45,46,47,48,49] suggests that the *GmaAMY* genes could be regulated during adaptation to biotic and/or abiotic stresses and in the growth of cotyledons and seeds. *GmaAMY1*–*GmaAMY3* and *GmaAMY5* could have differential expression during the development of SAM, and *GmaAMY1*, *GmaAMY2*, and *GmaAMY5*—during that of roots, nodules, and flowers, whereas *GmaAMY2* could change its mRNA levels during nitrogen fixation in nodules.

All predicted GmaAMY proteins contain the catalytic domain with the 3D structure, signatures, and active sites (Table 2, Figure 4, Supplementary Figure S2) characteristic of α-amylases (according to [31,50,51,54]), confirming that GmaAMY1–GmaAMY5 proteins should have α-amylase activity. GmaAMY1 and GmaAMY3 differ from the other GmaAMYs by the presence of two N-terminal starch-binding domains CBM45-1 and CBM45-2 (Supplementary Figure S3), which presumably mediate the interaction with the starch granule surface, as shown for AtAMY3 [56]. These results indicate that GmaAMY1 and GmaAMY3 may be specific for chloroplasts, have high affinity for starch molecules, and be functionally conserved and redundant.

Both GmaAMY1 and GmaAMY3 may form a disulfide bridge between two identified cysteine residues (Cys540/Cys628 and Cys534/Cys622, respectively), the *A. thaliana* analogs of which play a central role in the redox regulation of the AtAMY3 protein and are important for the structural stability and activity of the enzyme [55]. Formation of a disulfide bridge between the AtAMY3 residues Cys499 and Cys587 alters the tertiary structure and blocks the enzyme’s active site. Under oxidative conditions, the enzyme is activated through redox regulation by chloroplast thioredoxins [55]. Stress-induced inhibition of AtAMY3 activity can also be prevented by glutaredoxin. In this case, in addition to the Cys499 and Cys587, at least one more Cys residue can be oxidized/glutathionylated [63]. Presumably, such regulation of AtAMY3 occurs during stress responses [55], since under normal conditions, AtAMY3 does not affect transitory starch degradation [3]. However, after cold shock, *AtAMY3* gene expression increases, and *amy3* mutants accumulate more starch and less soluble sugars than wild-type plants [64]. The presence of similar cysteine residues in GmaAMY1 and GmaAMY3 suggests that their activity may also be redox-regulated when the plant is exposed to stress.

The phylogenetic position of GmaAMY2 (Figure 5) suggests probable functional differences with the proteins of the AtAMY3 subfamily, as evidenced by the presence of only one (Cys653) of the two cysteine residues critical for the redox regulation of chloroplast AMY3 in Arabidopsis [55]. The AtAMY3-Cys499 residue is generally conserved within the AtAMY3 subfamily, but in some plant species it is replaced by Leu, and another Cys, 11 amino acids upstream of Leu, may be involved in disulfide bridge formation [55]. However, GmaAMY2 has a Phe566 instead of the Cys499-analog and lacks upstream Cys residue.

Functional differences between GmaAMY2 protein and GmaAMY1 and GmaAMY3 are also follow from the atypical for α-amylases domain structure of the GmaAMY2 N-terminal part, including the absence of carbohydrate-binding modules (Figure 4, Supplementary Figure S3), similar to other AMY6-type α-amylases [17]. The N-terminal extension of GmaAMY2 contains two domains: one is Smc domain (structural maintenance of chromosomes) with ATPase activity and the other with unknown function (Table 2, Figure 4). We hypothesize that the detected structural mismatch may be due to either misassembly of the gene sequence or structural alterations during or after duplication. In the first case, it should be considered that *AMY6*-like genes have not been found in the Brassicaceae species [17]. The fact that the genome of the model species *A. thaliana* (Brassicaceae), with a thoroughly studied genome, does not contain an *AMY6*-like gene seems to be a strong argument for experimental validation of the presence of such a gene in the genome of different plant species. In the second case, neofunctionalization of GmaAMY2 can be assumed.

The effects of α-amylases on plant growth could vary across organs/tissues. Our analysis of soybean transcriptomes [58,59] revealed that *GmaAMY1–GmaAMY5* are actively expressed in the leaves and flowers (Figure 6), suggesting the role of soybean α-amylases in plant vegetative and reproductive development. Relatively high or moderate levels of *GmaAMY1–GmaAMY5* expression in pods (Figure 6) may be consistent with the requirement for starch breakdown products (soluble sugars) as an energy source for pod and seed development and maturation. Low levels of gene expression in seeds (Figure 6a) may correspond to starch accumulation in the endosperm of maturing seeds which enter a dormant state. High *GmaAMY1* and *GmaAMY2* transcription levels in roots and root hairs (Figure 6) suggest a role of these genes in root-specific carbohydrate metabolism, whereas the differential expression of *GmaAMY1*, *GmaAMY2* and *GmaAMY5* in infected roots (Figure 6a) may indicate their involvement in plant responses to biotic stress.

The interconversion between starch and soluble sugars plays one of the central roles in the adaptation of plants to abiotic stresses, including those with an osmotic component (drought, salinity, extreme temperatures) [65]. Drought or salinity has been shown to reduce starch content [12,28,29,30,31]. However, along with an increase in soluble sugar concentrations, we observed an increase in starch in response to all three stress types, with some exceptions (decrease in starch in the case of 2.5% PEG after 2 and 6 h of exposure, as well as during recovery from cold stress) (Figure 7 and Figure 8). It is possible that the increase in starch content we observed is associated with the short duration of stress (12 h cold, 24 h PEG or NaCl) in comparison with other studies, where carbohydrates are analyzed after prolonged exposure of 3 to 15 days [28,29,30,31]. Also, it should be noted that in study [28], the sharp decrease in starch content may also be due to the fact that the analyzed cotyledons were collected in the dark, when degradation of transient starch normally occurs. Furthermore, an increase rather than a decrease in starch content can be observed in the roots of stressed plants [28,29]. Finally, both the leaves and roots of five accessions of legume species *Vigna unguiculata* showed both an increase and a decrease (depending on the genotype) in starch in response to 16 days of 15% PEG-induced stress [66]. Thus, the response to osmotic stress is not necessarily accompanied by a rapid reduction in starch content. Stress-induced degradation may be delayed, as well as to be organ-specific or genotype-dependent. In addition, it is necessary to take into account the synthesis of transient starch that is still ongoing (during the daytime) in the leaves.

A study of two different drought-resistant tea plant varieties indicates that although genotype-dependent, the defense regulation mechanism based on carbohydrate metabolism in any case involves α-amylase activity [67]. It has been shown that prolonged drought leads to the upregulation of most *M. esculenta AMY* genes [12] and of the *G. max AMY3* gene [28,29]. In these studies, the activation of the *AMY* genes is accompanied by an increase in sugar content and a decrease in starch content. Other examples also confirm the role of α-amylases in plant stress response. Thus, in tea tree (*C. sinensis*), the expression of *CsAMY1* and *CsAMY2* is increased during the first hours of exposure to salinity, drought, and cold, whereas that of *CsAMY3* decreased [13]. In potato subjected to salinity and drought, the AtAMY3 subfamily genes *StAMY1* and *StAMY10* and the AtAMY1 subfamily gene *StAMY2* are upregulated, whereas the other AtAMY1 genes, *StAMY3* and *StAMY4*, are downregulated. The regulation of the AtAMY2 subfamily genes depends on the stress type; thus, during drought, potato *StAMY5* is activated and *StAMY6* and *StAMY7* are inhibited, whereas the opposite pattern is observed after salinity stress [11].

The *GmaAMY1–GmaAMY5* genes responded differently to soybean exposure to salinity, PEG-induced stress, and cold conditions (Figure 7 and Figure 8); a similar reaction of the *AMY* genes was observed in potato [11]. These results suggest that α-amylases may act synergistically to fine-tune starch degradation during adaptation of plants to adverse conditions.

We observed an increase in soluble sugar content in soybean after stress, which is consistent with the results of previous studies [12,28,29]. The amount of starch in plants subjected to salinity- and PEG-induced osmotic stress conditions also mainly increased (Figure 7 and Figure 8), which may be due to the short duration of the applied stresses and the delayed onset of starch degradation in the leaves, possibly because of the ongoing photosynthesis accompanied by the production of soluble sugars.

## 4. Materials and Methods

### 4.1. Identification and Structure Analysis of the G. max AMY Gene Family

The gene sequences recognized as α-amylases were searched in the *G. max* Williams 82 (Wm82) reference genome, single haplotype representation (Glycine max Wm82.a6.v1; release date 16 February 2024) (Phytozome; https://phytozome-next.jgi.doe.gov/info/Gmax_Wm82_a6_v1; assessed on 2 March 2026). The search was performed in Wm82.a6.v1 (Phytozome) proteome using BLASTP analysis (https://phytozome-next.jgi.doe.gov/blast-search, assessed on 2 March 2026, Expect (E) threshold < 0.01; comparison matrix BLOSUM62, which represents a clustering threshold of 62% identity) and the sequences of three known *Arabidopsis* α-amylases (AtAMY1, AtAMY2 and AtAMY3) as baits. Among the retrieved sequences, we selected only those encoding proteins containing the full-length α-amylase catalytic domain (the C-terminal half of the PLN02784 domain, which includes all known specific catalytic sites and signatures of α-amylase). NCBI accession numbers of these genes were determined using BLAST analysis (https://blast.ncbi.nlm.nih.gov/Blast.cgi; assessed on 10 April 2026; NCBI Glycine_max_v4.0, GCF_000004515.6). Chromosomal localization of the identified genes was visualized using MG2C v2.1 (http://mg2c.iask.in/mg2c_v2.1/; accessed on 15 April 2026 [68]), exon–intron structure was analyzed using GSDS v2.0 (https://gsds.gao-lab.org/; accessed on 15 April 2026 [69]), and intraspecies collinearity analysis was conducted in TBtools-II (v2.471) [70]. Promoter/5′-UTR-specific *cis*-regulatory elements were identified in the regions 2000 bp before the start codon using PlantCARE (http://bioinformatics.psb.ugent.be/webtools/plantcare/html/; accessed on 1 April 2026 [32]). Transcription factor (TF) binding sites were searched in the regions 2000 and 500 bp before the start codon using PlantTFDB (https://plantregmap.gao-lab.org/binding_site_prediction.php; accessed on 15 April 2026 [33]). Comparative nucleotide and amino acid sequence alignments were carried out in MEGA 7.0.26 [57]. MicroRNA targets in the *AMY* mRNA sequences were predicted using psRNATarget (plant small RNA target analysis server; https://www.zhaolab.org/psRNATarget/; accessed on 20 April 2026) with default parameters [38].

Bioinformatics analysis of the AMY protein structure was performed in terms of conserved domains (Conserved Domain Database; https://www.ncbi.nlm.nih.gov/cdd; accessed on 16 April 2026) and motifs (MEME 5.5.9; http://meme-suite.org/tools/meme; accessed on 23 April 2026), subcellular localization (SCL) (WoLF PSORT; https://wolfpsort.hgc.jp/; accessed on 16 April 2026), presence of a signal peptide (SP) (SignalP-6.0; https://services.healthtech.dtu.dk/services/SignalP-6.0/; accessed on 16 April 2026), tertiary structure (Phyre 2.2, https://www.sbg.bio.ic.ac.uk/phyre2/; accessed on 29 April 2026 [71]), and phylogenetic relationships (MEGA 7.0.26: Maximum Likelihood method, the JTT matrix-based model, bootstrap 1000). Phylogenetic tree was based on the amylase protein sequences of *G. max*, dicots *A. thaliana* (TAIR, https://www.arabidopsis.org/; accessed on 16 April 2026), *G. soja* (NCBI RefSeq assembly GCF_004193775.1), *V. unguiculata* (cowpea) (GCF_004118075.1), *M. truncatula* (barrel medic) (GCF_003473485.1), *P. vulgaris* (common bean) (GCF_000499845.2) and *L. japonicas* (GCF_012489685.1), and monocot *H. vulgare* (CAX51374_1, CAX51372.1) and *O. sativa* Japonica Group (Rice Genome Annotation Project, Release 7 (RGAP.r7); https://rice.uga.edu/; accessed on 16 April 2026; gene names are given according to [15]).

Physicochemical properties of putative *G. max* AMY proteins, such as molecular weight (MW), isoelectric point (pI), aliphatic index (AI), instability index (II), and grand average of hydropathy (GRAVY), were predicted using ExPASy ProtParam (https://www.expasy.org/resources/protparam; accessed on 16 April 2026). PANNZER2 (http://ekhidna2.biocenter.helsinki.fi/sanspanz/; accessed on 16 April 2026) was used for Gene Ontology (GO: biological process (BP) and molecular function (MF)) predictions.

### 4.2. In Silico Profiling of G. max AMY Gene Expression

Tissue/organ-specific expression patterns were determined in silico by analyzing transcriptomes in SoyTFBase (http://106.15.250.134:5000/, assessed on 24 April 2026), Gene Expression Resources for Soybean (GERS, https://www.soybase.org/tools/expression/, assessed on 24 April 2026), and Legume Information System (LIS, https://mines.legumeinfo.org/glycinemine/, assessed on 24 April 2026 [58,59]). RNAseq data given in TPM (transcripts per million) were presented as a heatmap constructed using Heatmapper 1.0 [72].

### 4.3. Modeling Abiotic Stresses for Soybean Plants

Seeds of soybean cv. Doka were germinated in water at 23–25 °C and a 16 h light/8 h dark cycle until active growth of roots and sprouts. Three week-old seedlings of 27–30 cm with three developed three-lobed leaves were exposed to the conditions of high salinity (100 mM NaCl) and PEG-induced drought (2.5, 5, 10, 15, and 20% PEG-6000) for 2, 6, and 24 h. Salt treatment for 24 h had no visible effect. In the case of 2.5% PEG, wilting of individual leaves and slight curling of the edges of the remaining leaves (most) were observed after 24 h. However, exposure to 5–20% PEG was lethal to the plants: severe wilting (6 h point) and death (24 h point) (Supplementary Figure S6).

Cold effects were tested by exposing five-week-old plants to 4 °C for 6 and 12 h and then allowing them to recover under normal conditions for 36 h. Exposure to low temperature for 12 h had no visible effect on the plants (Supplementary Figure S6).

The experiments were performed in three biological and three technical replicates; untreated plants grown under normal conditions were used as controls. Leaves were collected at each time point, frozen in liquid nitrogen, and stored at −80 °C until analysis.

### 4.4. Real-Time PCR (RT-qPCR) Analysis of AMY Expression

Total RNA was extracted from the leaves (RNeasy Plant Mini Kit and RNase-free DNase set; QIAGEN, Hilden, Germany) and used to synthesize cDNA (GoScript Reverse Transcription System; Promega, Madison, WI, USA). RT-qPCR was performed with 3.0 ng of cDNA, SYBR Green RT-qPCR mixture (Syntol, Moscow, Russia), SynTaq DNA polymerase (included in the SYBR Green RT-qPCR mixture; Syntol, Moscow, Russia), and cDNA-specific primers separated by one intron (Supplementary Table S3) in a CFX96 Real-Time PCR Detection System (Bio-Rad Laboratories, Hercules, CA, USA) at the following cycling conditions: 95 °C for 5 min and 40 cycles of 95 °C for 15 s and 60 °C for 40 s.

The expression of *AMY* genes was analyzed in three biological and three technical replicates and normalized to that of two stably expressed reference genes encoding elongation factor 1-beta (*GmaEF1B*; NCBI Gene ID LOC100500082) and hypothetical protein UKN2 (*GmaUKN2*; LOC100789577); these reference genes have demonstrated high expression stability under stress conditions such as salinity, drought, darkness and virus infection [73].

### 4.5. Analysis of Starch and Soluble Sugar Contents

Measurements were performed in the same leaf samples. To determine total starch content (mg/g of fresh weight), finely ground tissue (~0.5 g) was suspended in 4.5 mL of 33% (*v*/*v*) dimethyl sulfoxide and 0.44 M HCl, incubated at 60 °C for 30 min, cooled to room temperature, diluted 5-fold with MilliQ water, adjusted to pH 4.5 with 5 M NaOH, and filtered through Miracloth (Merck, Kenilworth, NJ, USA). The final solution (100 µL) was used to determine starch content with a STARCH kit (Boehringer Mannheim/R-Biopharm, Rotkreuz, Switzerland) according to the manufacturer’s protocol.

To analyze the content (mg/100 g FW) of soluble sugars (glucose, fructose, and sucrose), the Enzytec™ Liquid D-Glucose/D-Fructose and Enzytec™ Liquid Sucrose/D-Glucose kits (R-Biopharm AG, Darmstadt, Germany) were used according to the manufacturer’s instructions.

### 4.6. Statistical Analysis

RT-qPCR and biochemical data were statistically processed using GraphPad Prism v. 9 (https://www.graphpad.com/scientific-software/prism/; accessed on 27 April 2026) and visualized as graphics (GraphPad Prism v. 9) and heatmaps (Excel). The results were compared based on Two-Way ANOVA (multiple comparisons corrected with Bonferroni test; GraphPad Prism v. 9); *p* < 0.05 was considered to indicate a statistically significant difference.

## 5. Conclusions

We identified five soybean α-amylase-encoding genes, *GmaAMY1–GmaAMY5*, belonging to three α-amylase subfamilies: AtAMY1 (*GmaAMY5*), AtAMY2 (*GmaAMY4*), AtAMY3 (*GmaAMY1*, *GmaAMY3*), and AMY6 (*GmaAMY2*). *GmaAMY1–GmaAMY5* mRNAs were predicted to be targets of miRNAs associated with stress response, organ development, and nitrogen fixation in nodules. Two putative proteins of the AtAMY3 subfamily (GmaAMY1 and GmaAMY3) are close homologues of *A. thaliana* AMY3, and, thus, could have a role in starch degradation in chloroplasts and be regulated through redox potential. AMY6-subfamily GmaAMY2 forms a divergent branch within AtAMY3 as it lacks N-terminal starch-binding CBM45 domains, suggesting possible incorrect assembly of the gene 5′-end or gene neofunctionalization. GmaAMY5 may be a secretory α-amylase involved in starch degradation in the extracellular space. Under salinity, PEG-induced stress, and cold conditions, *GmaAMY1–GmaAMY5* genes exhibit differential expression with a distinct pattern for each, suggesting synergistic activity of α-amylases to fine-tune starch content regulation during adaptation of soybean to stress. Our results suggest that soybean amylases may play a role in plant defense by maintaining the necessary balance between starch degradation and soluble sugar accumulation. Identification and characterization of *GmaAMY* genes provide a basis for their further functional analysis and may facilitate the development of stress-tolerant soybean varieties.

## Figures and Tables

**Table 1:** Characteristics of the *G. max AMY* genes.

Gene	Gene ID ^1^	Genomic Localization ^1^	Gene, bp ^1^	Exon Number	CDS ^2^, bp
*GmaAMY1*	GLYMA.02G020500/100810333	Gm02:1842810..1850291 forward/chr2:1842892..1849421	7481/6530	13	2787
*GmaAMY2*	GLYMA.08G244900/100817238	Gm08:21251446..21263114 reverse/chr8:21212824..21223598	11,668/10,775	14	2874
*GmaAMY3*	GLYMA.08G296800/100788193	Gm08:43722143..43732053 forward/chr8:40703286..40712493	9910/9208	13	2769
*GmaAMY4*	GLYMA.14G222600/100813490	Gm14:52482349..52488314 forward/chr14:49619642..49624731	5965/5090	12	1242
*GmaAMY5*	GLYMA.17G242400/100815664	Gm17:42245810..42248646 forward/chr17:39920951..39923319	2836/2369	4	1245

Note: ^1^ according to Glycine max Wm82.a6.v1 (https://phytozome-next.jgi.doe.gov/info/Gmax_Wm82_a6_v1, accessed on 2 March 2026)/NCBI Glycine_max_v4.0 (GCF_000004515.6); ^2^ CDS—coding DNA sequences.

**Table 2:** Characteristics of putative GmaAMY proteins.

Parameter	GmaAMY1	GmaAMY2	GmaAMY3	GmaAMY4	GmaAMY5
MW, kDa	105.45	108.76	104.11	47.37	46.15
GO: BP/MF	GO:0005975 carbohydrate metabolic process/GO:0004556 α-amylase activity; GO:0005509 calcium ion binding				
SCL ^1^	Chlo/Cyto	Chlo	Chlo	Nucl	Chlo/Nucl/Extr/Vacu
pI	5.61	5.53	5.34	8.92	5.40
AI/II ^2^	74.36/42.46	84.23/45.70	74.99/38.36	66.54/41.63	79.90/31.31
GRAVY ^3^	−0.559	−0.502	−0.525	−0.623	−0.334
Size, aa	928	957	922	413	414
SP/TP; Conserved domain localization (aa)	No; PLN02784 (α-amylase, 1–928)	No; PLN02784 (α-amylase, 551–954) COG1196 (chromosome segregation ATPase Smc; cell cycle control, cell division, chromosome partitioning, 81–366) COG4372 (protein with DUF3084 domain, function unknown, 263–533)	No; PLN02784 (α-amylase, 7–922)	No; PLN02784 (α-amylase, 15–413)	Yes ^4^; PLN02784 (α-amylase, 25–414)
Active sites ^5^	Trp545, Tyr586, Phe671, Arg705, Phe708, Arg710, Trp734, Asp735 Asp707, Asp815, Glu732 (catalytic sites) His626, Lys718 (Ca binding) His626, His814 (substrate binding)	Trp571, Tyr611, Phe696, Arg730, Phe733, Arg735, Trp759, Asp760 Asp732, Asp839, Glu757 (catalytic sites) His651, Lys743 (Ca binding) His651, His838 (substrate binding)	Trp539, Tyr580, Phe665, Arg699, Phe702, Arg704, Trp728, Asp729 Asp701, Asp809, Glu726 (catalytic sites) His620, Lys712 (Ca binding) His620, His808 (substrate binding)	Trp34, Tyr74, Phe157, Arg191, Phe194, Lys196, Trp220, Asp221 Asp193, Asp300, Glu218 (catalytic sites) His114, Lys204 (Ca binding) His114, His299 (substrate binding)	Trp32, Tyr73, Tyr165, Arg199, Tyr202, Lys204, Trp228, Asp229 Asp201, Asp302, Glu226 (catalytic sites) His114, Lys 212 (Ca binding) His114, His301 (substrate binding)
Conserved signatures (I–IV) localization (aa) ^6^	DAVLNH (621–626) GWRLDFVRG (703–711) GEYWD (731–735) FIENHD (810–815)	DVVLNH (646–651) GWRLDFVRG (728–736) GEYWD (756–760) FLENHD (834–839)	DAVLNH (615–620) GWRLDFVRG (697–705) GEYWD (725–729) FIENHD (804–809)	DIVINH (109–114) DFRFDFVKG (189–197) GEYWD (217–221) FVDNHD (295–300)	DIVINH (109–114) GWRFDYVKG (197–205) GEKWD (225–229) FIDNHD (297–302)

Note: ^1^ chlo—chloroplast, cyto—cytoplasm, nucl—nucleus, extr—extracellular, vacu—vacuole; ^2^ AI = 65–85 indicates moderate content of aliphatic amino acids, characteristic of globular proteins, and II ≥ 40 corresponds to unstable proteins; ^3^ protein hydrophilicity corresponds to negative GRAVY scores; ^4^ N-terminal SP with cleavage site between positions 23 and 24 (probability 0.736632; putative secretory protein); ^5^ according to the NCBI-CDD and [34]; ^6^ according to [50,51]. Characteristics of putative GmaAMY proteins.

## Data Availability

The original contributions presented in this study are included in the article/Supplementary Materials. Further inquiries can be directed to the corresponding author.

## Supplementary Materials

The following supporting information can be downloaded at https://www.mdpi.com/article/10.3390/ijms27177915/s1.
